# Prevalence of Metabolic Syndrome and Its Components in Patients With Major Depressive Disorder

**DOI:** 10.7759/cureus.83066

**Published:** 2025-04-27

**Authors:** Asfand Yar Wali Faizan, Sheherzad Faizan, Sikandar Ilyas, Hammad Mudassar, Haseeba Khalid, Sidra Naseer, Iqra Naeem, Muhammad Zaryab Haider, Muhammad Irfan Jamil, Adeel Ahmed

**Affiliations:** 1 Medicine, Al Shifa Surgical Hospital, Dera Ghazi Khan, PAK; 2 Medicine, Ali Ahmad Khan Hospital, Jhang, PAK; 3 Anesthesia, Ali Ahmad Khan Hospital, Jhang, PAK; 4 Medicine, Akhar Saeed Medical and Dental College, Lahore, PAK; 5 Internal Medicine, Akhtar Saeed Medical and Dental College, Lahore, PAK; 6 Psychiatry and Behavioral Sciences, Lahore General Hospital, Lahore, PAK; 7 Psychiatry and Behavioral Sciences, University Medical and Dental College Faisalabad, Faisalabad, PAK; 8 Acute Internal Medicine, Blackpool Teaching Hospitals NHS Foundation Trust, Blackpool, GBR; 9 Cardiology, Rawalpindi Institute of Cardiology, Rawalpindi, PAK; 10 Nephrology, Lahore General Hospital, Lahore, PAK; 11 Medicine, Lahore General Hospital, Lahore, PAK

**Keywords:** hdl-c, lipid profile, major depressive disorder (mdd), metabolic syndrome (mets), obesity

## Abstract

Aim and background: This study aimed to determine the prevalence of metabolic syndrome (MetS) and its individual components among treatment-naïve patients with major depressive disorder (MDD) and to evaluate their association with depression severity among patients presenting to a tertiary care hospital. Understanding this relationship is essential, as coexisting MetS in patients with MDD contributes to elevated cardiovascular risk and complicates long-term psychiatric and medical outcomes.

Materials and methods: This cross-sectional study was conducted at the Department of Psychiatry, Lahore General Hospital, Lahore, Pakistan, from January 2023 to August 2024. A total of 290 treatment-naïve patients with MDD were enrolled using non-probability consecutive sampling. Diagnosis was based on the Diagnostic and Statistical Manual of Mental Disorders, Fifth Edition (DSM-5) criteria, and depression severity was assessed via the 17-item Hamilton Depression Rating Scale (HAM-D-17). MetS was defined using the National Cholesterol Education Program Adult Treatment Panel III (NCEP ATP III) criteria. Anthropometric and biochemical measurements were obtained under standardized protocols. Pearson correlation assessed associations between HAM-D scores and metabolic parameters. Binary logistic regression identified predictors of MetS, adjusting for age, sex, waist circumference, triglycerides, and HAM-D score. Statistical significance was set at p < 0.05.

Results: Out of 290 patients, the mean age was 37.39 ± 10.78 years; 181 (62.4%) were female and 109 (37.6%) were male. MetS was present in 81 (27.9%) patients. Central obesity was identified in 102 (35.2%), hypertension in 104 (35.9%), hypertriglyceridemia in 89 (30.7%), impaired fasting glucose in 88 (30.3%), and low high-density lipoprotein cholesterol (HDL-C) in 42 (14.5%). At least one metabolic abnormality was noted in 193 (66.6%) participants. Mean HAM-D scores were significantly higher among patients with MetS (19.42 ± 4.11) than those without (16.93 ± 4.44; p < 0.001). Patients with central obesity (102, 35.2%) had higher depression scores (19.42 ± 3.82 vs. 16.65 ± 4.52; p < 0.001), as did those with hypertriglyceridemia (19.79 ± 4.15 vs. 16.67 ± 4.30; p < 0.001), hypertension (18.69 ± 4.65 vs. 17.03 ± 4.29; p = 0.003), and impaired fasting glucose (19.05 ± 4.26 vs. 17.00 ± 4.45; p < 0.001). No significant difference was observed for low HDL-C (p = 0.829). Chi-square analysis revealed significant associations between the severity of depression and all MetS components except HDL-C (p < 0.05). On binary logistic regression, male gender (adjusted odds ratio (AOR) = 0.019; p = 0.019), increasing age (AOR = 1.083; p = 0.011), triglyceride level (AOR = 1.017; p = 0.028), and HAM-D score (AOR = 1.176; p = 0.005) were significant predictors of MetS.

Conclusion: MetS and its components were commonly observed among patients with MDD and were significantly associated with depression severity. Triglycerides, age, and depression scores were independent predictors of MetS. These findings highlight the importance of routine metabolic screening and integrated care approaches for individuals with depression to reduce cardiovascular risk and improve overall clinical outcomes.

## Introduction

Major depressive disorder (MDD) is recognized as a highly prevalent and disabling psychiatric illness, currently affecting around 280 million people globally. The World Health Organization (WHO) identifies MDD as a leading contributor to global disability, with projections indicating it will become the most burdensome mental disorder by 2030 [1]. Data from the Global Burden of Disease project demonstrate a continuous increase in its position based on disability-adjusted life years (DALYs), underscoring its significant public health relevance [2].

Growing research highlights a bidirectional association between MDD and metabolic syndrome (MetS), a constellation of metabolic disturbances that includes increased waist circumference, elevated blood pressure, raised fasting glucose, hypertriglyceridemia, and reduced levels of high-density lipoprotein cholesterol (HDL-C). These conditions are believed to be linked through common biological pathways, such as hypothalamic-pituitary-adrenal (HPA) axis dysfunction, persistent low-grade inflammation, oxidative stress, and impaired insulin sensitivity [3,4].

Several international studies have documented a higher prevalence of MetS in patients with depressive disorders compared to the general population [5-7]. Estimates vary significantly depending on geographic region, diagnostic criteria, population demographics, and treatment exposure, with reported prevalence rates ranging from 20% to 45% [5,6,8,9]. Central obesity, hypertriglyceridemia, and low HDL-C are the most frequently encountered components among these patients. The severity of depressive symptoms, particularly in recurrent or chronic forms of MDD, has also been associated with greater metabolic burden [5,6]. Furthermore, existing evidence suggests that metabolic disturbances may be present even in drug-naïve individuals, implying that factors intrinsic to the depressive disorder itself, independent of psychotropic medications, may contribute to MetS pathogenesis [10-12].

Despite growing interest in the MDD-MetS comorbidity, most previous research has either focused on the prevalence of depression in patients already diagnosed with MetS or evaluated the effects of antidepressant medications on metabolic parameters [5,6,8]. Few studies have specifically examined the frequency of MetS and its individual components in patients diagnosed with MDD, particularly in those who are treatment-naïve [7,11]. This gap in the literature is even more pronounced in lower-middle-income countries, where regional data are limited and local clinical screening practices often do not integrate metabolic assessment into psychiatric evaluations. In Pakistan, published data comprehensively exploring the prevalence of MetS and their clinical correlates among treatment-naïve patients with MDD remain scarce. The present study was conducted to determine the hospital-based prevalence of MetS and its individual components among treatment-naïve MDD patients and to evaluate the relationship between depression severity and metabolic abnormalities.

## Materials and methods

This hospital-based descriptive cross-sectional study was conducted to determine the prevalence of MetS and its individual components in treatment-naïve patients diagnosed with MDD. The study was conducted at the Department of Psychiatry, Lahore General Hospital, Lahore, Pakistan. Data collection was carried out from January 2023 to August 2024. The approval for the study was taken from the ethical review committee (IRB # 126/01/2023). A sample size of 290 patients was calculated using the WHO sample size calculator, based on an expected 25% prevalence of MetS in MDD, with a 95% confidence level and 5% margin of error [5]. Participants were selected using a non-probability consecutive sampling method. Both inpatient and outpatient individuals aged between 18 and 60 years, newly diagnosed with MDD or previously diagnosed but untreated for the past three months, were included. Exclusion criteria included current use of psychotropic drugs, substance use, pregnancy, or history of chronic metabolic, endocrine, or cardiovascular disorders. Written informed consent was taken from all the participants.

The diagnosis of MDD was established by a trained psychiatrist based on the Diagnostic and Statistical Manual of Mental Disorders, Fifth Edition (DSM-5), using a structured clinical interview [13]. Depression severity was evaluated through the 17-item Hamilton Depression Rating Scale (HAM-D-17), a standardized clinician-rated tool. Depression severity was assessed using the HAM-D-17, a standardized clinician-rated tool with established validity and reliability. Patients were categorized based on HAM-D-17 total scores: mild depression (8-16), moderate depression (17-23), and severe depression (≥24), as per standard cutoffs [14]. Appropriate psychiatric support, counseling, and referral for treatment were provided to all patients diagnosed with MDD to ensure ethical management, and none were left unattended after evaluation. The primary outcome was the presence of MetS, assessed according to the National Cholesterol Education Program Adult Treatment Panel III (NCEP ATP III) criteria, which defines MetS as the presence of any three or more of the following five components: (1) central obesity (waist circumference >102 cm in men or >88 cm in women), (2) elevated triglycerides (≥150 mg/dL), (3) reduced HDL-C (<40 mg/dL in men or <50 mg/dL in women), (4) elevated blood pressure (≥130/85 mmHg or use of antihypertensive medication), and (5) elevated fasting plasma glucose (≥100 mg/dL or diagnosis of diabetes mellitus) [15].

Demographic and clinical data, including age, sex, marital status, education, body mass index (BMI), blood pressure, waist circumference, and fasting laboratory values, were collected. Laboratory investigations included fasting blood glucose, serum triglycerides, and HDL-C, assessed after an overnight fast. Blood pressure was measured using a calibrated device after 10 minutes of rest, and waist circumference was assessed using a non-elastic tape midway between the lowest rib and iliac crest. Fasting venous blood was drawn after an overnight fast and processed in the hospital’s biochemistry lab under standardized quality protocols.

Statistical analysis was performed using IBM SPSS Statistics version 26.0 (IBM Corp., Armonk, NY). Quantitative variables were reported as mean ± standard deviation, while qualitative data were presented as frequency and percentage (n%). Comparisons between groups with and without MetS were analyzed using the independent samples t-test for qualitative data and the quantitative variables for categorical variables. Pearson correlation was used to assess the relationship between HAM-D scores and metabolic variables. Binary logistic regression was applied to determine predictors of MetS, adjusting for age, sex, waist circumference, triglycerides, and HAM-D score. A p-value less than 0.05 was considered statistically significant.

## Results

A total of 290 patients were enrolled (mean age: 37.39 ± 10.78 years); 181 (62.4%) were female and 109 (37.6%) were male. Age groups included 92 (31.7%) aged between 18 and 30 years, 118 (40.7%) aged between 31 and 45 years, and 80 (27.6%) aged between 46 and 60 years. Marital status of the participants was as follows: 147 (50.7%) were married, 100 (34.5%) were unmarried, and 43 (14.8%) were divorced/widowed. Mean systolic/diastolic blood pressure was 124.25 ± 14.67/79.65 ± 8.35 mmHg; BMI was 25.24 ± 3.23 kg/m²; waist circumference was 81.56 ± 10.23 cm; fasting glucose was 92.57 ± 20.10 mg/dL; triglycerides were 133.69 ± 39.38 mg/dL; and HDL-C was 52.93 ± 9.51 mg/dL. Central obesity was found in 102 (35.2%) participants, hypertriglyceridemia in 89 (30.7%), low HDL-C in 42 (14.5%), hypertension in 104 (35.9%), and impaired fasting glucose in 88 (30.3%) participants. MetS was present in 81 (27.9%), while 193 (66.6%) had ≥1 abnormality and 97 (33.4%) had none. Depression severity per HAM-D was as follows: 51 (17.6%) cases were mild, 114 (39.3%) were moderate, 85 (29.3%) were severe, and 40 (13.8%) cases were very severe.

The comparison of demographic, clinical, and biochemical characteristics between patients with and without MetS revealed statistically significant differences in several key variables, including age, systolic and diastolic blood pressure, fasting blood glucose, triglycerides, HDL-C, and depression severity as measured by HAM-D scores. However, no significant differences were observed in BMI or waist circumference. Detailed metrics and statistical comparisons are presented in Table 1.

**Table 1:** Comparison of clinical, metabolic, and demographic parameters between MDD patients with and without MetS (n = 290). Continuous variables are presented as mean ± standard deviation (SD) and compared using independent samples t-tests, reported as t(df), p-value, and 95% confidence interval (CI) of the mean difference. Categorical variables are presented as frequency and percentage (n%), and analyzed using the chi-square test (χ²) with degrees of freedom (df). MetS: metabolic syndrome; SD: standard deviation; CI: confidence interval; BP: blood pressure; BMI: body mass index; HDL-C: high-density lipoprotein cholesterol; HAM-D: Hamilton Depression Rating Scale

Variable	Categories/units	MetS present (n = 81)	MetS absent (n = 209)	Test statistic (t / χ²)	p-value	95% CI
Age (years)	Mean ± SD	45.11 ± 9.32	34.39 ± 9.80	t= 8.47	<0.001	8.23–13.21
Age group	18–30 years	10 (12.3%)	82 (39.2%)	χ² = 62.48	<0.001	-
	31–45 years	22 (27.2%)	96 (45.9%)			
	46–60 years	49 (60.5%)	31 (14.8%)			
Gender	Male	13 (16.0%)	96 (45.9%)	χ² = 22.22	<0.001	-
	Female	68 (84.0%)	113 (54.1%)			
Marital status	Married	43 (53.1%)	104 (49.8%)	χ² = 12.40	0.002	-
	Unmarried	18 (22.2%)	82 (39.2%)			
	Divorced/Widowed	20 (24.7%)	23 (11.0%)			
Systolic BP (mmHg)	Mean ± SD	133.90 ± 14.76	120.51 ± 12.84	t = 7.63	<0.001	9.94–16.85
Diastolic BP (mmHg)	Mean ± SD	84.85 ± 7.94	77.64 ± 7.62	t = 7.15	<0.001	5.23–9.20
BMI (kg/m²)	Mean ± SD	24.91 ± 2.44	25.36 ± 3.48	t = 1.07	0.287	–1.28–0.38
Waist circumference	Mean ± SD (cm)	79.89 ± 7.93	82.20 ± 10.94	t = 1.73	0.084	–4.94–0.31
Fasting glucose	Mean ± SD (mg/dL)	115.58 ± 16.36	83.65 ± 13.13	t = 17.30	<0.001	28.30–35.57
Triglycerides	Mean ± SD (mg/dL)	154.47 ± 35.85	125.64 ± 37.77	t = 5.91	<0.001	19.24–38.43
HDL-C	Mean ± SD (mg/dL)	50.99 ± 11.56	53.68 ± 8.50	t = 2.18	0.030	–5.13––0.26
HAM-D score	Mean ± SD	19.42 ± 4.11	16.93 ± 4.44	t = 4.38	<0.001	1.37–3.61
HAM-D severity	Mild	4 (4.9%)	47 (22.5%)	χ² = 19.04	<0.001	-
	Moderate	30 (37.0%)	84 (40.2%)			
	Severe	28 (34.6%)	57 (27.3%)			
	Very Severe	19 (23.5%)	21 (10.0%)			

Patients with central obesity had higher HAM-D scores (19.42 ± 3.82 vs. 16.65 ± 4.52; p < 0.001; r = 0.296), as did those with hypertriglyceridemia (19.79 ± 4.15 vs. 16.67 ± 4.30; p < 0.001; r = 0.321). Impaired fasting glucose (19.05 ± 4.26 vs. 17.00 ± 4.45; p < 0.001; r = 0.210) and hypertension (18.69 ± 4.65 vs. 17.03 ± 4.29; p = 0.003; r = 0.178) also showed significant associations. No significant difference was observed for low HDL-C (p = 0.829). Detailed metrics are provided in Table 2.

**Table 2:** Comparison of HAM-D scores and correlation with metabolic abnormalities among patients (n = 290) Comparison of Hamilton Depression Rating Scale (HAM-D-17) scores between patients with and without metabolic abnormalities using independent sample t-test, along with Pearson correlation (r) to assess the strength and direction of linear association. A p-value < 0.05 was considered statistically significant. HDL-C: high-density lipoprotein cholesterol

Metabolic parameter	Yes (Mean ± SD)	No (Mean ± SD)	t-value	p-value	95% CI of difference	Correlation (r)
Central obesity	19.42 ± 3.82	16.65 ± 4.52	-5.52	<0.001	-3.76 to -1.78	0.296
Hypertriglyceridemia	19.79 ± 4.15	16.67 ± 4.30	-5.84	<0.001	-4.17 to -2.07	0.321
Low HDL-C	17.50 ± 3.90	17.65 ± 4.58	0.22	0.829	-1.19 to 1.48	-0.011
Hypertension	18.69 ± 4.65	17.03 ± 4.29	-3.01	0.003	-2.76 to -0.57	0.178
Impaired fasting glucose	19.05 ± 4.26	17.00 ± 4.45	-3.64	<0.001	-3.13 to -0.95	0.210
Metabolic syndrome	19.42 ± 4.11	16.93 ± 4.44	-4.53	<0.001	-3.58 to -1.41	0.250

Chi-square analysis revealed significant associations between depression severity and most metabolic parameters. Central obesity (χ² = 25.999, p < 0.001), hypertriglyceridemia (χ² = 19.268, p < 0.001), hypertension (χ² = 17.897, p < 0.001), and impaired fasting glucose (χ² = 15.254, p = 0.002) were all significantly related to more severe depression categories. In contrast, low HDL-C was not significantly associated with depression severity (p = 0.130). Complete frequency details are summarized in Table 3.

**Table 3:** Association between metabolic syndrome components and HAM-D depression severity categories (n = 290) The chi-square (χ²) test was used to assess the statistical significance of the association between depression severity and each metabolic factor. A p-value < 0.05 was considered statistically significant. HDL-C: high-density lipoprotein cholesterol; HAM-D: Hamilton Depression Rating Scale

Metabolic variable	Category	Mild (n=51)	Moderate (n=114)	Severe (n=85)	Very Severe (n=40)	χ² (df)	p-value
Central obesity	Yes	4 (3.9%)	38 (37.3%)	40 (39.2%)	20 (19.6%)	25.999 (3)	<0.001
	No	47 (25.0%)	76 (40.4%)	45 (23.9%)	20 (10.6%)		
Hypertriglyceridemia	Yes	6 (6.7%)	30 (33.7%)	33 (37.1%)	20 (22.5%)	19.268 (3)	<0.001
	No	45 (22.4%)	84 (41.8%)	52 (25.9%)	20 (10.0%)		
Low HDL-C	Yes	5 (11.9%)	23 (54.8%)	8 (19.0%)	6 (14.3%)	5.658 (3)	0.130
	No	46 (18.5%)	91 (36.7%)	77 (31.0%)	34 (13.7%)		
Hypertension	Yes	11 (10.6%)	36 (34.6%)	32 (30.8%)	25 (24.0%)	17.897 (3)	<0.001
	No	40 (21.5%)	78 (41.9%)	53 (28.5%)	15 (8.1%)		
Impaired fasting glucose	Yes	7 (8.0%)	30 (34.1%)	32 (36.4%)	19 (21.6%)	15.254 (3)	0.002
	No	44 (21.8%)	84 (41.6%)	53 (26.2%)	21 (10.4%)		
Metabolic syndrome	Yes	4 (4.9%)	30 (37.0%)	28 (34.6%)	19 (23.5%)	19.041 (3)	<0.001
	No	47 (22.5%)	84 (40.2%)	57 (27.3%)	21 (10.0%)		

A binary logistic regression model was applied to identify predictors of metabolic syndrome. The model was statistically significant (χ² = 97.35, p < 0.001) and correctly classified 83.4% of the cases. Significant predictors included male gender, which was inversely associated with MetS (adjusted odds ratio (AOR) = 0.019, 95% CI: 0.002-0.173, p = 0.019). Age was a positive predictor (AOR = 1.083, 95% CI: 1.019-1.151, p = 0.011), indicating increasing odds of MetS with advancing age. Waist circumference showed a borderline statistically significant association (AOR = 1.072, 95% CI: 0.993-1.158, p = 0.074). Triglycerides (AOR = 1.017, 95% CI: 1.002-1.032, p = 0.028) and HAM-D scores (AOR = 1.176, 95% CI: 1.048-1.320, p = 0.005) were also significant predictors, suggesting that both metabolic and psychiatric severity parameters contribute to MetS risk.

## Discussion

In this cross-sectional study of 290 patients with MDD, MetS was observed in 27.9%, reflecting a notable comorbid burden. MetS was significantly more prevalent in female patients than male patients (84.0% vs. 16.0%; χ² = 22.22, p < 0.001) and in patients aged between 46 and 60 years (60.5%; χ² = 62.48, p < 0.001). Patients with MetS had higher HAM-D scores (19.42 ± 4.11 vs. 16.93 ± 4.44; p < 0.001) and more often exhibited severe or very severe depression (58.1% vs. 37.3%). These findings suggest that greater depressive severity is associated with increased metabolic dysregulation, consistent with prior studies [16].

The prevalence of MetS observed in this study (27.9%) is consistent with previously reported rates ranging from 25% to 50% among individuals diagnosed with MDD, depending on population characteristics and diagnostic definitions. Comparable findings were reported by Agarwal et al. (2016), who identified a MetS prevalence of 26% among patients with recurrent depression in India [5]. A meta-analysis by Vancampfort et al. (2015) similarly estimated a pooled prevalence of 30.5% in MDD patients, while a systematic review from Ethiopia reported a prevalence of 37.3% among those with severe mental illness [8,17]. These results underscore the global burden of MetS in psychiatric populations. The present findings are also in agreement with those of Ljubičić et al., who observed higher MetS rates in recurrent versus first-episode MDD [7]. Although depressive episodes were not stratified in the current study, greater MetS frequency in older participants suggests illness chronicity. Furthermore, consistent with prior literature, fasting glucose, triglycerides, and HDL-C were the most frequently altered parameters, with depression severity significantly associated with MetS presence [5,6,18].

The predominance of female patients among those with MDD and MetS observed in this study aligns with previous findings. In the present sample, 84% of MDD patients with MetS were female, and female sex was associated with approximately fourfold higher odds of MetS. A similar pattern was reported by Moorthy et al., where nearly 80% of depressed individuals with MetS were women, highlighting gender as a significant risk factor [16]. Proposed mechanisms include greater susceptibility to central obesity, hormonal transitions during menopause, and medication-related weight gain in women. However, some studies, including Ljubičić et al., suggest that once illness duration is considered, sex differences may attenuate [7]. The meta-analysis from Ethiopia also reported higher odds of MetS in females (AOR ≈ 2.66), although not statistically significant, possibly due to limited sample size [8]. These findings support the view that women with MDD may require enhanced metabolic monitoring.

In the present study, central obesity was identified in 102 (35.2%) patients with MDD, making it one of the most common metabolic abnormalities. This aligns with findings by Agarwal et al., who observed central obesity in a significant proportion of depressed patients and demonstrated its association with HAM-D scores [5]​. A moderate positive correlation (r = 0.296, p < 0.001) between waist circumference and HAM-D scores further supports this association. Hypertension was present in 104 (35.9%) patients and was significantly associated with depression severity (χ² = 17.897, p < 0.001). These findings are consistent with global trends [4,19]​. Kao et al. reported a heightened risk of hypertension in MDD, with hazard ratios over 2.5 in some subgroups [19].

Hypertriglyceridemia was detected in 89 (30.7%) participants, and those affected had higher HAM-D scores (19.79 ± 4.15 vs. 16.67 ± 4.30; p < 0.001), with a strong correlation (r = 0.321). Zhu et al. also found hypertriglyceridemia more frequent in MDD patients with impaired glucose regulation [20]. Moreira et al. reported significantly higher triglyceride levels among depressed individuals compared to controls [21]. In contrast, low HDL-C was found in only 42 (14.5%) patients and did not show a significant correlation with HAM-D severity (p = 0.829), consistent with earlier studies reporting variable associations [20]. Impaired fasting glucose was observed in 88 (30.3%) cases and was significantly associated with both higher HAM-D scores (19.05 ± 4.26 vs. 17.00 ± 4.45, p < 0.001) and depressive severity (χ² = 15.254, p = 0.002), in line with findings by Liu et al., who linked hyperglycemia with greater psychiatric burden [22]. The shared biological mechanisms between MDD and metabolic dysregulation involve several interconnected pathways. Hyperactivity of the HPA axis is one of the central mechanisms, resulting in sustained cortisol elevation, which contributes to abdominal obesity, insulin resistance, and lipid abnormalities [23]. Moreover, disturbances in glucose-insulin homeostasis have been observed among depressed patients. Impaired insulin sensitivity and beta-cell dysfunction are believed to underlie the association between depression and hyperglycemia, further increasing the risk of MetS [24].

Significant associations were observed between depressive severity and multiple metabolic parameters. Patients with central obesity, hypertriglyceridemia, and impaired fasting glucose had significantly higher HAM-D scores (p < 0.001), with moderate correlations (r = 0.296 to 0.321). Hypertension also showed a significant association (p = 0.003), while low HDL-C did not. A Mendelian randomization study further supports depression as a causal risk factor for metabolic syndrome, reinforcing the importance of early psychiatric and metabolic intervention [4].

This study highlights important clinical implications. The observed association between depression severity and MetS underscores the need for early identification of metabolic risks in patients with MDD. Routine screening for metabolic abnormalities in psychiatric settings could allow earlier intervention, helping to reduce long-term cardiovascular and metabolic complications. Integrated management strategies combining depression treatment with lifestyle modifications, such as diet and exercise, may provide greater benefits than treating each condition separately. These findings support the need for collaborative care models and suggest that multidisciplinary approaches targeting both mental and physical health may improve overall patient outcomes. This study’s strengths include a relatively large sample size, systematic assessment of all MetS components using validated NCEP ATP-III criteria, and objective evaluation of depression severity via the HAM-D scale. Inclusion of both inpatients and outpatients across a wide age range (18-60 years) enhances generalizability. The exclusive focus on confirmed MDD cases allows better specificity in assessing associations. However, its cross-sectional design limits causal inference. Absence of a healthy control group and unadjusted confounders (e.g., lifestyle, medication type) constrain interpretability. Selection bias may exist due to hospital-based recruitment, and the use of standard, non-ethnic-specific MetS thresholds may underreport central obesity. Future research should focus on longitudinal studies to clarify the causal link between depression and MetS. Randomized controlled trials integrating lifestyle interventions with depression treatment are recommended to assess dual benefits. Biomarker-based studies exploring inflammation, HPA axis dysfunction, and metabolic risk in MDD are needed. Investigations into medication-related metabolic effects, especially in high-risk groups like middle-aged women, are also warranted. Implementation of collaborative care models with routine MetS screening in psychiatric settings could enhance long-term health outcomes for patients with major depression.

## Conclusions

This study identified a high burden of metabolic abnormalities among patients with MDD, with MetS present in over a quarter of participants. Significant associations were observed between depression severity and multiple metabolic components, particularly hypertriglyceridemia, impaired fasting glucose, hypertension, and central obesity. Patients with more severe depressive symptoms were substantially more likely to exhibit metabolic derangements. Age, male gender, triglyceride levels, and depression severity independently predicted the presence of MetS. These findings emphasize the need for integrated screening and management of both mental and physical health parameters in depressed individuals to prevent long-term cardiometabolic consequences.
